# Telehealth Diabetes Prevention Intervention for the Next Generation of African American Youth: Protocol for a Pilot Trial

**DOI:** 10.2196/25699

**Published:** 2021-03-31

**Authors:** Abigail Gamble, Bettina M Beech, Breanna C Wade, Victor D Sutton, Crystal Lim, Shanda Sandridge, Michael A Welsch

**Affiliations:** 1 Department of Preventive Medicine John D Bower School of Population Health University of Mississippi Medical Center Jackson, MS United States; 2 Department of Pediatrics School of Medicine University of Mississippi Medical Center Jackson, MS United States; 3 Myrlie Evers-Williams Institute for the Elimination of Health Disparities John D Bower School of Population Health University of Mississippi Medical Center Jackson, MS United States; 4 Department of Health Systems and Population Health Science College of Medicine University of Houston Houston, TX United States; 5 Office of Preventive Health and Health Equity Mississippi State Department of Health Ridgeland, MS United States; 6 Division of Psychology Department of Psychiatry and Human Behavior University of Mississippi Medical Center Jackson, MS United States; 7 Pediatric Gastroenterology Children's Healthcare of Mississippi Jackson, MS United States; 8 Department of Population Health Science John D Bower School of Population Health University of Mississippi Medical Center Jackson, MS United States

**Keywords:** prediabetic state, child obesity, telehealth, obesity management, behavioral science, implementation science, Jackson Heart Study, Centers for Disease Control and Prevention, preventive medicine, mobile phone

## Abstract

**Background:**

In 1999, type 2 diabetes mellitus (T2DM) was identified as an emerging epidemic in youth, and racial and ethnic minority youth were identified with high risk. Two decades later, no gold standard T2DM prevention intervention has been established for this population.

**Objective:**

This study tests the efficacy of a telehealth diabetes prevention intervention for African American (AA) families with children with risk for T2DM. Concurrently, investigators aim to evaluate an implementation strategy for the uptake of the intervention by the University of Mississippi Medical Center’s (UMMC) pediatric weight management clinic.

**Methods:**

This single-arm trial will enroll 20 parents with overweight or obesity of children (8-11 years) with overweight or obesity, both of whom are at risk for T2DM. Parents will meet in small groups (5 parents per group) weekly for 11 weeks and then monthly for 4 monthly maintenance sessions via videoconference using Wi-Fi–enabled iPads with cellular connectivity. The intervention will be adapted from the National Diabetes Prevention Program and *Power to Prevent*, a diabetes prevention program tailored for AA families. The same lifestyle intervention facilitated by a racially concordant lifestyle coach trained in the Diabetes Prevention Program will be delivered to all groups (n=4). Participants will be recruited in-person during patient encounters at the UMMC’s pediatric weight management clinic. Sessions will consist of dietary and physical activity behavior change strategies facilitated using problem-solving and goal-setting skills. The implementation strategy has 2 targets: the pediatric weight management clinic site and clinical team and parents of children at risk for T2DM engaged in intensive obesity treatment to prevent T2DM. The multifaceted implementation protocol includes 4 discrete strategies: creating a new clinical team, changing the service site, intervening with families, and promoting organizational readiness for change.

**Results:**

Recruitment and enrollment began in December 2020, and the intervention is scheduled to be delivered to the first cohort of parents in March 2021. The results are expected to be submitted for publication beginning in November 2021 through 2022. The primary outcome measure for the pilot trial will include changes from baseline to 12 and 30 weeks in the child BMI *z* score and parent BMI. The implementation evaluation will include multiple measures of feasibility, acceptability, appropriateness, fidelity, and efficacy. This protocol was approved by the UMMC’s Institutional Review Board (#2020V0249).

**Conclusions:**

The proposed intervention approach is supported by the scientific literature and is scalable given the current and future health care subsidies for telehealth. Findings from this pilot trial will begin to address critical barriers to defining a gold standard lifestyle intervention for AA families with children at risk for T2DM. If effective, the intervention could be feasibly disseminated to treat obesity and prevent T2DM in high-risk AA pediatric populations.

**International Registered Report Identifier (IRRID):**

PRR1-10.2196/25699

## Introduction

### Background

In 1999, Rosenbloom and Winter [1] labeled type 2 diabetes mellitus (T2DM) in youth as an emerging epidemic, identifying racial and ethnic minority groups, whose proportion in the US population continues to increase [2], at highest risk. T2DM is a chronic state of hyperglycemia that clusters in families because of genetic, cultural, behavioral, and environmental risk factors [3,4]. In 2018, an estimated 32.4 million (10.5%) people of all ages in the United States had T2DM, and an additional 88 million adults (34.5%; ≥18 years) and 1 in 5 youths (12-18 years) were estimated to have had prediabetes [5,6]. Predictions indicate that with rising incidence rates, the proportion of US youth (<20 years) with T2DM may quadruple between 2010 (22,820 youths) and 2050 (84,131 youths [7]), and African American (AA) youth represent a vulnerable pediatric population with high risk [7,8].

Alarmingly, T2DM presents a more severe and aggressive disease course in youth than in adults [9], and there is no optimal treatment for T2DM in youth [10]. In a retrospective analysis of youth and adults with obesity and impaired glucose tolerance, adolescents experienced more severe insulin resistance than adults with similar adiposity and glycemic status [9]. Of particular concern are the results from the recent RISE (Restoring Insulin Secretion) Pediatric Medication Study that evaluated whether β-cell function and metabolic control could be effectively altered by a pharmacological intervention [11]. Results from this randomized controlled trial with 91 pubertal youths (10-19 years) with obesity and existing impaired glucose tolerance randomized to 12 months of metformin showed that the early pharmacologic intervention was ineffective in slowing disease progression [11]. These findings underscore the need for strategies to prevent or delay the onset of T2DM in youth at high risk [12].

Prediabetes is a precursor to T2DM, characterized by hyperglycemic parameters below the diabetes threshold [13,14]. An estimated 5% to 10% of prediabetes cases advance to T2DM per annum [15], and according to the American Diabetes Association (ADA), up to 70% of people with prediabetes will progress to T2DM at some point in their lifetime [16]. Although prediabetes may never progress to T2DM, unfavorable cardiometabolic profiles remain among people with prediabetes, putting them at increased risk for cardiovascular-related morbidities and premature mortality [17]. Consequently, youth with prediabetes are faced with earlier and prolonged exposure to myriad cardiometabolic abnormalities, accentuating the critical need for obesity treatment in the primary prevention of T2DM in youth [7,10].

### Diabetes Prevention

Studies have indicated that adults with prediabetes can mitigate T2DM prognosis by taking preventive action, such as participation in the National Diabetes Prevention Program (DPP) [18]. The DPP began as a multisite randomized controlled trial, which demonstrated the effectiveness of a 12-month intensive lifestyle intervention over pharmaceutical treatment for preventing or delaying T2DM among adult participants with prediabetes across all racial and ethnic groups [19]. At 2.8- and 10-year follow-up, participants randomized to the lifestyle intervention group reduced their risk of developing T2DM by 58% and 34% compared with 31% and 18% in the metformin group, respectively [20,21]. In 2010, US Congress authorized the Centers for Disease Control and Prevention to lead the dissemination of the DPP [18] as a targeted strategy and population approach to reduce the incidence of T2DM among adults with prediabetes [22].

Intensive lifestyle modification is the recommended treatment for pediatric patients with prediabetes [23]; however, there is no gold standard lifestyle intervention to prevent T2DM in youth. Previous studies have demonstrated the feasibility of lifestyle interventions for youth with overweight or obesity and those at risk for T2DM and the importance of tailored interventions for racial and ethnic minority groups; however, limited efficacy has been shown in reducing T2DM risk and improving obesity outcomes [24-26]. Intervention studies with youth have also been hindered by the insufficient representation of racial and ethnic minority youth at high risk for T2DM in obesity treatment trials [27]. *Power to Prevent* is a diabetes prevention intervention from the Centers for Disease Control and Prevention based on the DPP and tailored for AA families [28]. To our knowledge, this intervention has not yet been evaluated among AA families with children at risk for T2DM or disseminated through health care institutions. Given the restrictions on in-person gatherings because of the COVID-19 pandemic and the consistent literature reporting high attrition rates among in-person interventions, there is a need for novel and sustainable implementation modalities for delivering lifestyle interventions to racial and ethnic minority families at high risk for T2DM [24].

Telehealth has demonstrated effectiveness in delivering obesity and prediabetes treatment [29-33] and has mitigated barriers to accessing care for millions of Americans [31]. In particular, videoconferencing has been widely used to deliver effective T2DM care for adult and young patients [33-36] and has recently established efficacy for group-based interventions [37-39]. In a review of 17 lifestyle intervention studies, the authors found that tablet-based group videoconferencing was feasible, accessible, and acceptable, regardless of participants’ digital literacy (technology skills) [37]. Across studies, videoconferencing groups cultivated cohesiveness and bonding similar to in-person settings, and participants developed behavioral skills including lifestyle behaviors to treat obesity and demonstrated improved health outcomes along with high attendance rates (66%-93.8%) and few dropouts [37]. Intervention group sizes averaged from 5 to 7 participants and met weekly for 1 hour over 12 to 26 sessions in total [37]. The use of telehealth technology to deliver a family-based lifestyle diabetes prevention intervention tailored for AA families is likely to offer a scalable approach to reduce T2DM among high-risk youth [40-42].

### Objectives

The Telehealth Diabetes Prevention Intervention for the Next Generation of African American Youth (TELE-GEN) pilot trial proposes implementing and evaluating a diabetes prevention intervention based on the *Power to Prevent* program for AA parents of children (8-11 years) who are both overweight or obese and at risk for T2DM. Historically, a lengthy effectiveness study conducted under *ideal circumstances* would be the next step in identifying a gold standard lifestyle intervention to prevent T2DM in AA youth; however, standard effectiveness studies are limited by their inability for rapid translation and the need for subsequent implementation studies to validate utility in real-world settings [43]. Hybrid research designs leverage the rigor of clinical trials and implementation science to foster rapid translation and effective implementation [33,43]. Thus, in partnership with the Center for Telehealth at the University of Mississippi Medical Center (UMMC) and pediatric weight management clinic, we propose a novel effectiveness-implementation hybrid type II research design [43] with the coprimary aim to (1) conduct a 30-week, single-arm pilot trial with at-risk parents of at-risk children to assess the early efficacy of Power to Prevent delivered using small group-based videoconferencing to reduce BMI in parents (n=20) and stabilize or reduce BMI z scores in children (n=20), while (2) conducting a comprehensive evaluation of a multifaceted implementation strategy for the uptake of the intervention by the pediatric weight management clinic at the UMMC in Jackson, Mississippi. The protocol described below was approved by the UMMC’s Institutional Review Board (#2020V0249).

## Methods

### Study Setting

The Diabetes Belt is a distinct geographic region in the southern United States where county-level adult T2DM rates (≥11%) are especially high and obesity and physical inactivity account for one-third of all diagnosed cases [44]. Mississippi is the only state in which every county (82 counties) is represented by the Diabetes Belt. According to America’s Health Rankings 2019 Report, Mississippi had the third-highest adult T2DM rate (14.3%) in the United States, followed by the second highest adult obesity (39.5%) and physical inactivity (32%) rates [45]. Recent data from the National Survey of Children’s Health (2018-2019) reported in the State of Obesity Report, 2020 [46], identified Mississippi as having the second-highest youth (10-17 years) obesity rate (22.3%) nationally. In addition, AA adults (45.7% [45]) and youth (25.4% [47]) had higher rates of obesity compared with their White counterparts (36.3% [45] and 21.7% [47], respectively). Mississippi also has the highest percentage of Black population (37.8% [48]), and 42% of Mississippi youth are Black compared with 14% nationally [49]. Mississippians experience persistently high rates of poverty (19.7%) and child poverty (27%) [48], and the state is mostly rural and medically underserved [50]; social and environmental factors contribute to poor health and disease. In this context, AA youth in Mississippi are among those at highest risk for T2DM.

The UMMC is the state’s only academic medical center and has the only children’s hospital in the state. In 2019, 4464 AA children aged 8 to 11 years were treated by a UMMC pediatric clinic, and between November 2019 and April 2020, 170 AA children aged 8 to 11 years were identified with the risk for T2DM in the UMMC’s Prediabetes Registry. UMMC is a minority-serving, research-intensive health care organization with a state-mandated requirement to provide not less than 50% of their services to indigent persons (Mississippi Legal Code Annotated 37-115-27; 2017). The UMMC is also a national leader in telehealth [51] and has one of only 2 National Centers of Excellence in Telehealth in the nation [52]. The UMMC Center for Telehealth has successfully intervened with adults with uncontrolled T2DM [53] using remote patient monitoring and is piloting a similar single-arm feasibility trial with pediatric patients (10-17 years) [54]. Health systems with telehealth technology are encouraged to implement and evaluate weight loss interventions for patients at risk for T2DM [32,40,55], and Mississippi is at the forefront of advancing telehealth and adopting mandates for telehealth care reimbursement [51-53,56]. Thus, Mississippi is an ideal location to test the effectiveness and implementation of lifestyle interventions using telehealth in clinical care settings to treat obesity and prevent T2DM in AA youth at the highest risk for diabetes in the United States.

### Participant Recruitment and Sample Size

In-person participant screening and enrollment will be conducted in the UMMC pediatric weight management clinic beginning in December 2020. Preliminary screening will the conducted by the clinical provider using a patient eligibility checklist that will screen for both child and parent eligibility. The lifestyle coach will immediately meet in the clinic room following the clinical encounter to conduct a full screening and provide detailed study information to interested parents and children. Consent forms will be verbally described to parents and children, and parent consent, parent permission, and child assent will be obtained before study enrollment.

Children will be eligible to participate if they are AA, overweight or obese (≥85th percentile for age- and sex-specific BMI), and aged between 8 and 11 years. The child age range was selected to allow for intervention occurring before the average age of youth-onset T2DM, 13 years [10]. The criteria for parents (biological, grandparent, or legal guardian) of eligible children included AA, overweight or obese (BMI ≥25 kg/m^2^), and coresidence with the child. Although mothers represent most participants in family-based studies, we will not use parents’ sex as an inclusion criterion. Parents and children must also be at risk for T2DM based on the following criteria: (1) parents’ T2DM risk will be assessed using the A1CNow^+^ system to measure point-of-care hemoglobin A_1c_ (HbA_1c_), which uses a drop of nonfasting blood (5.7%-6.4%), and the ADA prediabetes screening tool (score ≥5); (2) child risk will be based on the BMI percentile (≥85th percentile) and parents’ risk (BMI ≥25 and ADA screening or HbA_1c_).

Consistent with previous T2DM prevention interventions [26,57], we will exclude families if the child or parent has a history of T2DM or a screening HbA_1c_ level greater than 6.4%, is taking glucose-lowering medications, is participating in a supervised weight loss program, is pregnant or breastfeeding, has conditions limiting participation, has undergone weight loss surgery, or is moving out of state within 6 months. If a parent becomes pregnant during the study, we will include only child data (index participant) in the analyses. Technology literacy will not be included as an exclusion criterion. Participants will receive technology instruction from the telehealth center and be provided technical support as needed.

The power calculation, across 5 scenarios, shows detectable effect sizes, ≥−2.5 (SD 0.05) BMI in parents [58] and in children, where a modest reduction in BMI z score (≥0 to <−0.1, SD 0.05) has been associated with improved cardiometabolic risk [59]. In the first 3 scenarios, assuming 10% attrition [37], a minimum of 18 dyads are needed. In scenarios 4 and 5, detectable effect sizes are reported in the case of 0% (n=20 dyads) and 20% (n=16 dyads) attrition. We anticipate 20% attrition, which is consistent with the group- and family-based telehealth lifestyle intervention literature [60-62]. Analyses are based on α=.05 and a conservative two-sided test. An effect size of 1.0 is considered substantial and 0.5 is considered medium. We will obtain reliable effect size estimates to design a pilot randomized controlled trial.

### Conceptual Model

**Figure 1:**
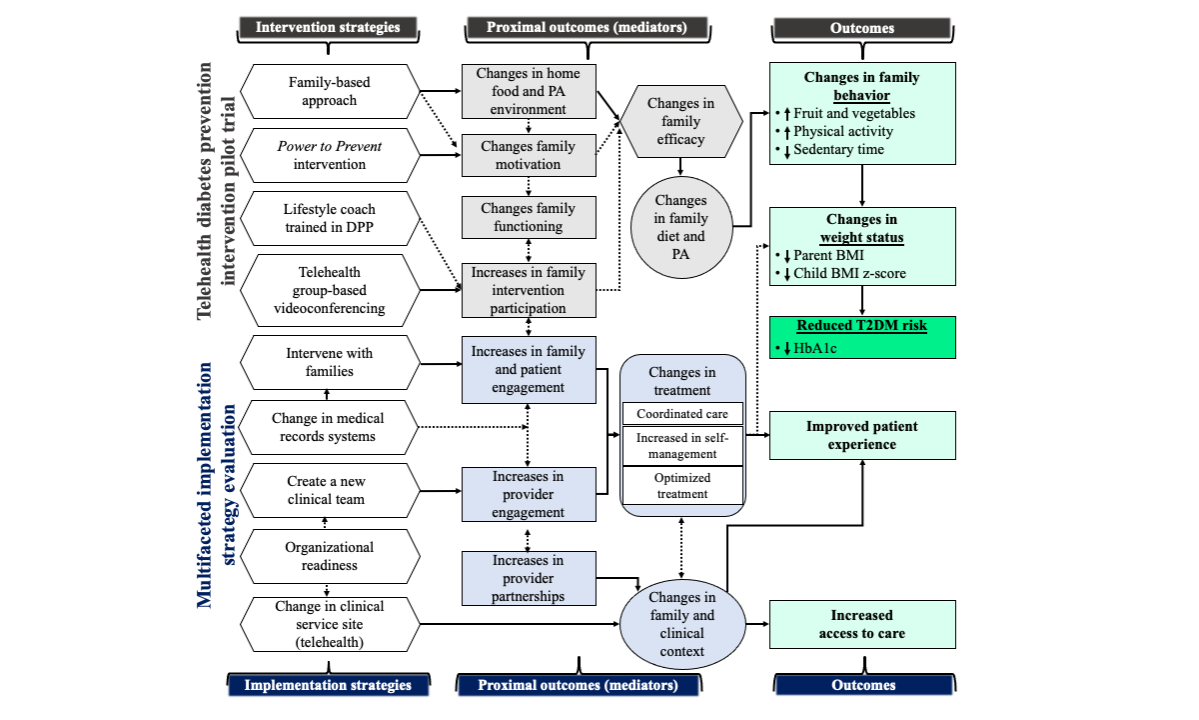
Telehealth diabetes prevention intervention and implementation conceptual model. DPP: Diabetes Prevention Program; PA: physical activity.

Family Systems Theory, undergirded by family motivation and Social Learning Theory, and the Telehealth in Chronic Disease (TECH) model are the foundations for the TELE-GEN conceptual model (Figure 1). Social Learning Theory is used to guide intensive lifestyle interventions, such as the DPP [63]. The conceptual basis for the TELE-GEN intervention has evolved from pediatric obesity and T2DM prevention research using Family Systems Theory [64,65] and Social Learning Theory [66,67]. Successful Social Learning Theory–based intervention approaches include helping families make changes to the home food, physical activity, and screen time environment, increasing family motivation to make positive changes, and increasing family functioning around food, physical activity, and sedentary behaviors. These activities are thought to increase family efficacy for making lifestyle modifications and ultimately improving intervention outcomes. This theoretical approach is bolstered by applying the TECH model, which outlines effective pathways for the uptake of clinical telehealth interventions [68]. The TELE-GEN conceptual model is displayed in Figure 1.

### Intervention

Power to Prevent [28] is a diabetes prevention intervention based on the DPP [63] and the *Small Steps, Big Rewards Program* [69] and is tailored for AA families. The goal of the program is to achieve a 5% to 7% weight loss by making modifications to dietary, physical activity, and sedentary behaviors. In the proposed pilot trial, modification to family dietary, physical activity (≥150 min per week), and sedentary behaviors will be targeted, in addition to individual-level parent behaviors. The *Power to Prevent* *Leader Guide* and participant materials will be adapted and used in each session. The scientific literature strongly supports family-based approaches to treating youth with overweight or obesity [70]. Thus, group-based sessions (60 min) will be conducted with parents and target the adoption, maintenance, and self-regulation of family-focused dietary, physical activity, and sedentary behaviors. Parent participants will meet weekly for 11 weeks and then monthly for 4 pilot maintenance sessions (15 sessions in total; frequency and duration supported by the literature [71]). Sessions for all groups will be facilitated by a racially concordant lifestyle coach (interventionist) who will be trained in accordance with the Centers for Disease Control and Prevention Diabetes Prevention Recognition Program standards [72]. Wi-Fi–equipped iPads (12.9”) with cellular connectivity will be used to link families to videoconferencing sessions and to cooking and family-friendly physical activity video demonstrations via YouTube.

#### Intervention Targets

In 2013, an obesity expert panel reviewed 146 lifestyle intervention studies and reported a high degree of evidence for the effectiveness of diet with caloric restriction and increased physical activity for weight loss in adults [73]. Behavioral interventions to treat obesity are deemed safe for youth (4-18 years); however, fostering healthy eating behaviors is recommended over caloric restriction for weight loss in youth [74]. Thus, the dietary targets for children are to increase fruit and vegetable consumption, reduce sugar-sweetened beverage intake, and adopt appropriate portion sizes. The dietary targets for parents are to reduce the overall caloric intake by reducing the consumption of sugar-sweetened beverages and dietary fat and to increase fruit and vegetable intake. According to the 2018 Physical Activity Guidelines [75] and the 2009 American College of Sports Medicine Position Statement [76], the family (parent and child) physical activity goal is 60 minutes of moderate-to-vigorous activity (3 or more metabolic equivalents) per day on 5 or more days per week. In addition, based on the recommendations for treating obesity and reducing T2DM risk in youth, the sedentary target will be to reduce out-of-school time watching television, playing video games, and/or using a tablet, iPad, or smartphone to less than 2 hours per day.

#### Intervention Sessions

Group sessions will consist of identifying behavior change strategies leading to achieving the intervention targets (20 minutes), problem solving and decision making to circumvent barriers (20 minutes), and family goal setting and action planning (20 minutes). Parents will be coached to self-select behavior change strategies and practice skills that will enable them to do things such as making healthy foods available in the home, modifying recipes to be healthier, reading food labels, increasing family physical activity, and creating boundaries for the use of technology to reduce out-of-school sedentary time. Parents will be encouraged to use behavioral reinforcement strategies with their children, such as giving immediate praise, creating realistic expectations, and rewarding effort [77]. The practice of brainstorming will be used to encourage parents as a collective group to self-discover solutions to behavior change barriers and support participants in achieving their goals. Brainstorming has proven to be an effective component of problem solving and mitigating barriers to sustainable behavior change [78-82]. Action planning will be used to facilitate parents in setting realistic, attainable, and short-term (weekly) goals for making dietary and physical activity changes. Goal setting positively correlates with weight loss [83], and action planning is an effective self-monitoring strategy that will allow participants to personalize their goals and gradually achieve intervention targets [84,85].

#### Intervention Participant Retention

Telehealth is an effective retention strategy used in the DPP [86] and mitigates transportation barriers [87]. The lifestyle coach will dedicate time outside of intervention sessions to build rapport with parents and will function as a personal contact for participants to establish meaningful relationships, send meeting reminders, and schedule and conduct make-up sessions (consistent with DPP [63]) [86]. Participants will be incentivized using attendance raffles for cooking (eg, measuring cups), physical activity equipment (eg, jump rope), and incremental monetary compensation at each assessment point (baseline, US $50; postintervention, US $75; follow-up, US $150) [86]. Study assessments will be conducted in-person at the time of the child’s routine appointment with the pediatric weight management clinic and monetary resources will be allocated to families experiencing transportation barriers.

#### Intervention Efficacy Measures

Primary and secondary outcome measures will be assessed at baseline (week 1), postintervention (week 12), and 4-month follow-up (week 30). The primary outcome measure will be a reduction or stabilization in the child BMI *z* score (index participant) and a reduction in parent BMI (coparticipant) [88]. Secondary outcomes include anthropometric (waist circumference and body composition), biomarker (HbA_1c_, blood pressure, resting heart rate, lipid profile, and Tanner stage), behavioral (fruit and vegetable, sugar-sweetened beverage, and sweat and salty snack consumption; family meal frequency; fast food meal frequency; physical activity volume; and sedentary time), psychosocial (self-efficacy, self-regulation, readiness to change, family functioning, and perceived stress), and environmental (home food and physical activity environment and perceived neighborhood support for healthy eating and physical activity) measures (Table 1). All the measurements will be recorded electronically using REDCap (Research Electronic Data Capture) tools hosted at the UMMC [89]. REDCap is a secure, web-based software platform designed to support data capture for research studies, providing an intuitive interface for validated data capture; audit trails for tracking data manipulation and export procedures; automated export procedures for seamless data downloads to common statistical packages; and procedures for data integration and interoperability with external sources.

**Table 1:** Pilot trial outcome measures.

Outcome measure and instrument				Participant
**Anthropometric measures**				
	**BMI *z* score**			
		Stature (0.1 cm) assessed using Seca stadiometer, and weight (0.1 kg) assessed using Tanita (TBF-400) body composition and weight scale; height and weight measured without shoes; BMI *z* score using a CDC^a^ SAS program for the 2000 growth charts ages 0 to <20 years [90]	Child	
	**BMI**			
		Stature (0.1 cm) assessed using Seca stadiometer, and weight (0.1 kg) assessed using Tanita (TBF-400) body composition and weight scale; height and weight measured without shoes; BMI calculated using the CDC formula: weight (kg)/(height [m])^2^ [91]	Parent	
	**Body composition**			
		Tanita (TBF-400 Body Composition Monitor) uses bioelectrical impedance to assess fat and fat-free mass	Parent and child	
	**Waist circumference**			
		Seca 203 ergonomic circumference measuring tape	Parent and child	
**Biomarker measures**				
	**HbA_1c_^b^**			
		A1CNow^+^ system [92]; patient-friendly, point-of-care fingerstick with 5 µl blood (nonfasting)	Parent and child	
	**Lipid profile**			
		CardioChek Plus Analyzer [93]; patient-friendly, point-of-care fingerstick with 15-40 µl blood; measures total cholesterol, high-density lipoprotein, and triglycerides	Parent and child	
	**Systolic blood pressure**			
		IntelliSense professional digital blood pressure monitor using the American Heart Association guidelines for children and adults [94]	Parent and child	
	**Diastolic blood pressure**			
		IntelliSense professional digital blood pressure monitor using the American Heart Association guidelines for children and adults [94]	Parent and child	
	**Resting heart rate**			
		IntelliSense professional digital blood pressure monitor using the American Heart Association guidelines for children and adults [94]	Parent and child	
**Psychosocial measures**				
	**Self-efficacy**			
		Parental self-efficacy, 14 items (Norman et al [95]); subscales: confidence for encouraging healthy behaviors and role modeling healthy behaviors	Parent	
		Healthy Eating and Physical Activity Self-Efficacy Questionnaire, 10-items (Lassetter et al [96]); modified to assess a sedentary environment (2 items)	Child	
	**Home physical and social environment**			
		Modified Home PA and Food Environment Scale, 100 items (Gattshall et al [97]); subscales: availability and accessibility of food, physical activity equipment, and technology devices; parent role modeling and family policies for food, physical activity, and technology use	Parent	
	**Motivational readiness to change**			
		Family Motivational Readiness, 8 items (Rhee et al [98,99]); modified to assess technology and all questions with regard to child and family, 16 items total; subscales: helping child and family eat healthy, physical activity, and limit technology	Parent	
	**Family functioning**			
		McMaster Family Assessment Device, 60 items (Epstein et al [100]; Halliday et al [101]); subscales: problem solving, communication, roles, affective responsiveness, affective involvement, behavior control, and general functioning	Parent	
	**Neighborhood perceptions**			
		Neighborhood Perceptions Scale, 31 items (Mujahid et al [102]); subscales: aesthetic quality, walking environment, food availability, safety, violence, social cohesion, and activities with neighbors	Parent	
	**Stress**			
		Perceived Stress Scale, 10 items (Cohen et al [103])	Parent	
		PROMIS Pediatric Psychological and Physical Stress Scale, 19 items and 26 items (Bevans et al [104])	Child	
**Behavioral measures**				
	**Child behavior (subjective)**			
		Child Obesity Behavior Questionnaire; 20 items to measure parents’ perception of the child’s dietary, physical activity, and sedentary behaviors (Rhee et al [98])	Parent	
		Healthy Eating and Physical Activity Recall Questionnaire, 9 items (Lassetter et al [96]), modified to assess technology environment, and 5 items	Child	
	**Physical activity volume (objective)**			
		ActiGraph GT9X Accelerometer; device worn on the wrist of the nondominant hand for 7 consecutive days to measure volume of moderate-to-vigorous physical activity minutes (greater than 3 metabolic equivalents)	Parent and child	
	**Sedentary time (objective)**			
		ActiGraph GT9X Accelerometer; device worn on the wrist of the nondominant hand for 7 consecutive days to measure volume of low-intensity activity minutes (less than 3 metabolic equivalents)	Parent and child	
**Other measures**				
	**Pubertal stage**			
		Pubertal Development Scale, 5 items (Carskadon and Acebo [105])	Parent and child	

^a^CDC: Centers for Disease Control and Prevention.

^b^HbA_1c_: hemoglobin A_1c_.

### Data Analysis

Participant sociodemographic characteristics at baseline will be described using means and SDs, medians and ranges, or frequencies with percentages where applicable. The primary outcome evaluation will assess the change in child BMI *z* score (index participant) and parent BMI (coparticipant) from baseline to 12 and 30 weeks using repeated measures analysis of covariance with child Tanner stage as the covariate. Secondary analyses will include the comparison of anthropometric, biomarker, behavioral, psychosocial, and environmental measures adjusted for other factors. Generalized linear modeling will be used to assess the effect of the intervention, both unadjusted and adjusted (controlling for demographics, baseline BMI *z* score, and BMI). Data will be analyzed according to intention-to-treat principles [106-108].

### Implementation Strategy

The proposed implementation strategy has 2 targets: (1) the UMMC’s pediatric weight management clinical setting and clinical care team, which will be modified to support the uptake of the intervention and integration of the lifestyle coach into the clinical care team and (2) parents and children with T2DM risk referred to and engaged in intensive obesity treatment for the prevention of T2DM. The multifaceted implementation plan includes 4 discrete strategies: (1) creating a new clinical team, (2) changing the service site, (3) intervening with families, and (4) assessing organizational readiness [109]. In accordance with the recommendations for reporting implementation protocols [110], each strategy is operationalized in Table 2.

**Table 2:** Operationalization of discrete implementation strategies.

Strategy components	Discrete strategy			
	Create a new clinical team	Change service sites	Intervene with families	Readiness for change
Actor	Nurse practitioner; psychologist; lifestyle coach	Lifestyle coach; parent groups	Lifestyle coach; parent groups	Nurse practitioner; psychologist; lifestyle coach
Action	Nurse practitioner referral to lifestyle coach for screening and enrollment	Intervention delivered via videoconference; lifestyle support mirroring clinical treatment	Lifestyle coach provides support and conducts weekly outreach with parents	Identify barriers and facilitators, and processes impacting intervention uptake
Target	Integrate lifestyle coach as part of the clinical care team	Mitigate barriers to participation and engage the parent in treatment	Mitigate barriers, retention, and engagement	Health system and clinic workflow context
Dose	Continuous	Weekly (×11) and monthly (×4)	Point of referral to follow-up	Continuous
Outcome	Acceptability; feasibility; appropriateness	Acceptability; feasibility; appropriateness; fidelity; efficacy	Acceptability; feasibility; appropriateness; fidelity; efficacy	Acceptability; feasibility; appropriateness
Justification	American College of Physicians supports clinical care teams [111]; team-based lifestyle care for diabetic patients has been effective [112]	TECH^a^ model [68]; telehealth is practical given the COVID-19 pandemic [113]; an effective retention strategy [86] and mitigates transportation barriers [87]; videoconference is feasible, accessible, and acceptable [37]	Family Systems Theory [64,65]; Social Learning Theory [66,67]; TECH model [68]	Consolidated Framework for Implementation Research [114,115]

^a^TECH: Telehealth in Chronic Disease.

#### Implementation Evaluation

An iterative formative and process evaluation will be conducted to explore the complex processes, dynamic context, and organizational influences on implementation [116]. A mixed methods realist approach will be used to determine the extent to which the intervention was delivered as intended and whether the implementation strategy was acceptable, appropriate, and feasible [117]. Similar complex designs have been used to evaluate the implementation of lifestyle interventions with new populations and within new settings [116,118-121].

#### Evaluation Participants

The pediatric nurse practitioner, clinical psychologist, lifestyle coach (herein referred to as *providers*), and parent participants (herein referred to as *families*) will be asked to participate in our evaluation. The principal investigator (PI) will concurrently, independently, and iteratively collect quantitative and qualitative data throughout the trial. Data collection instruments will be developed in REDCap [89] and iPad accessible. Intervention sessions and study team meetings will be conducted and recorded using Big Blue Button, an open source web conferencing system. Attendance and outreach records will be recorded in a password-protected Microsoft Excel file.

#### Evaluation Data Sources

Five data sources will be used to gather sufficient evaluation data. Brief qualitative interviews will be conducted with providers (across and within waves) and families (within waves) at 3 time points: (1) during the intervention, (2) at postintervention, and (3) at follow-up. Family interviews during the intervention will be conducted by the PI via phone at the families’ convenience, and postsession and follow-up interviews will be conducted in-person during study assessments at the clinic. Provider interviews will be conducted by the PI via Big Blue Button on the UMMC campus. Interviews will probe providers’ and families’ perceptions of the acceptability, feasibility, and appropriateness of the intervention and the impact of telehealth on patient and provider engagement, provider partnerships, treatment outcomes, and access to care. Providers and the PI will also meet weekly for 30 minutes via Big Blue Button to review protocols, discuss implementation progress, identify barriers and solutions, and create action plans for adaptations to improve recruitment, retention, and intervention delivery.

Parents and the lifestyle coach will be asked to complete brief postsession surveys using their iPads to assess the acceptability and appropriateness of the intervention content, materials, and delivery. The lifestyle coach will be asked to document participation, the *Power to Prevent* resources that were used, and session components that were delivered and be asked to describe what worked well and the obstacles encountered. The lifestyle coach will record formal attendance and outreach encounters to document participant attendance at each core session and maintenance session, make-up sessions, and any outreach encounters via email, phone, or text message. Session observations will be conducted by the PI using an observation guide during one-third of all sessions to assess implementation fidelity, family engagement, and interactions among families and the lifestyle coach (context).

#### Evaluation Measures

Five fidelity dimensions will be evaluated, including adherence, exposure, quality of delivery, participant responsiveness, and intervention differentiation [122]. Data sources will include brief postsession surveys, session observations, and attendance and outreach records. Four feasibility dimensions will be evaluated, including recruitment, retention, engagement, clinical operations, and scheduling. Data sources will include interviews, surveys, session observations, attendance and outreach records, and meeting notes. Five acceptability dimensions will be evaluated, including changes in the clinical context and clinical team, changes in treatment, intervention materials, frequency and intensity of the intervention, and delivery via telehealth. Interviews and surveys will garner families’ perceptions of the intervention, how their views evolved during the intervention, and opinions of the maintenance phase. Providers’ and families’ perceptions of the importance and benefit of the intervention, impact on access to and engagement in the intervention, optimization of obesity treatment, patients’ experiences, and family and provider engagement will be assessed via interviews, surveys, and outreach and encounter records.

### Data Analysis

Qualitative meeting notes and clinical and intervention observations will be reviewed and synthesized on an ongoing basis to make immediate adaptations to screening and enrollment procedures, implementation strategies, and intervention session delivery and facilitation. All other evaluation data analyses will be conducted in 3 phases. In phase 1, qualitative and quantitative data sources will be independently analyzed. In phase 2, we will use a side-by-side comparison convergence approach where the qualitative findings are outlined and then converged with the respective quantitative results and efficacy outcomes for synthesis. In phase 3, the interpretation of findings, we will seek to understand if and how our strategy worked, for who, and in what context.

## Results

Recruitment and enrollment began in December 2020, and the first cohort of families will begin the intervention in March 2021. As of March 15, 2021, 10 children and their parents were enrolled in the study; however, intervention delivery had not begun. Future outcomes will be published in peer-reviewed health-related research journals and presented at national, regional, or state professional meetings and conferences. Preliminary findings will position the investigative team to design a subsequent hybrid study that will include a powered pilot randomized controlled trial with a larger sample of families and providers, a usual care control arm, and long-term follow-up; a robust evaluation of a refined implementation protocol; and cost-benefit and return-on-investment analyses.

## Discussion

The TELE-GEN pilot trial will begin to address critical barriers to defining a gold standard lifestyle intervention for AA families with children at risk for T2DM. The proposed approach is supported by the scientific literature [33,123] and leverages existing infrastructure to engage AA parents in a virtual community, a context most relevant in response to the current COVID-19 global pandemic [124-127]. The proposed study aims and innovative hybrid research design will contribute to advancing translational research by optimizing clinical and implementation science to improve health and reduce disease occurrence [128]. The proposed intervention is also scalable given the current health care subsidies for telehealth interventions [51,129,130] and future subsidies emerging from telehealth demands resulting from the COVID-19 pandemic [124-127]. Finally, the proposed intervention could be feasibly disseminated, adapted, and implemented to treat obesity and prevent T2DM in high-risk AA pediatric populations throughout the United States.

## Appendix

Multimedia Appendix 1 NIH Peer Review Summary Statement; 2019 COBRE submission.

Multimedia Appendix 2 NIH Peer Review Summary Statements; COBRE Submission 2020.

## Abbreviations

AA: African American
ADA: American Diabetes Association
DPP: Diabetes Prevention Program
HbA_1c_: hemoglobin A_1c_
PI: principal investigator
REDCap: Research Electronic Data Capture
T2DM: type 2 diabetes mellitus
TECH: Telehealth in Chronic Disease
TELE-GEN: Telehealth Diabetes Prevention Intervention for the Next Generation of African American Youth
UMMC: University of Mississippi Medical Center
