# Supplementary material for: Telehealth Diabetes Prevention Intervention for the Next Generation of African American Youth: Protocol for a Pilot Trial
Source: JMIR Res Protoc. 2021 Mar 31;10(3):e25699. doi: 10.2196/25699 (PMC8047807; doi:10.2196/25699)
Supplement: Multimedia Appendix 1 [file resprot_v10i3e25699_app1.pdf]

Budget and Period of Support:

Recommended budget modifications or possible overlap identified:

- It was not clear there were funds for a telehealth nurse.

Project-004 - Telehealth Diabetes Prevention Intervention for the Next Generation of African American Youth (TELE-GEN Study) (Description as provided by applicant): The Telehealth Diabetes Prevention Intervention for the Next Generation of AA Youth (TELE-GEN) study will implement and evaluate a telehealth diabetes prevention intervention with high risk African American children (6- to 11-years-old) and their parents, while comparing the efficacy of parent-and-child versus parent-only intervention approaches. Power to Prevent is a lifestyle diabetes prevention intervention from the Centers for Disease Control and Prevention that is based on the Diabetes Prevention Program and designed specifically for African American families. To our knowledge, this intervention has not yet been evaluated in a randomized trial with African American families with children at risk for T2DM, nor been delivered via telehealth. Our specific aims are to: (1) Conduct a randomized controlled pilot study to obtain preliminary evidence of the comparative efficacy of two telehealth diabetes prevention interventions for African American families (N=40) from which to design a future full-scale randomized controlled trial. (2) Determine the mediating effects of familial environmental and psychosocial variables on the primary outcome. Our primary outcome measure will be stabilization or reduction in BMI z-score in children (index participant) and a reduction in BMI in parents (co- participant). Eligible children and their parent(s) will be randomized to either a parent-and-child (n=20 families) or parent-only (n=20 families) arm and both will receive the telehealth intervention delivered by lifestyle coaches trained in the Diabetes Prevention Program. Families will participate in group-based sessions focused on the adoption and maintenance of positive dietary and physical activity behaviors. Families in each arm will meet weekly for 11-weeks (60-minute sessions), and then monthly (60-minute sessions) for 4 pilot behavioral reinforcement maintenance sessions (15 sessions total). Participants will meet in their respective groups (n=5 families per group) via videoconference using a tablet for the entire intervention. Sessions will consist of: an informational education portion focused on nutrition and physical activity (20 minutes), problem solving and decision-making skills to circumvent barriers to behavioral change (20 minutes), and family goal setting and action planning (20 minutes). The topics of the 11-core sessions will be the Power to Prevent intervention. Parents and children randomized to the parent-and-child arm and parents randomized to the parent-only arm will be asked to participate in each videoconference session. Assessment measures will be collected from the child and parent participants in both intervention arms at baseline, 12-weeks (post-intervention), and 30-weeks (follow-up). At the completion of this pilot study, we will be uniquely positioned to: 1) estimate sample size and logistics for a more robust TELE-GEN study with a larger sample of families and multiple pediatric providers in Mississippi, and 2) conduct a randomized controlled trial to assess the efficacy of a potentially sustainable and widely applicable intervention that addresses the T2DM burden among African American families.

CRITIQUE 1

SCORE (1-9): 5

Overall Impact: African-American (AA) youth who are prediabetic with high risk factors are more likely to progress to having diabetes. Research indicates that there is a lack of specific primary prevention intervention for African-American children; thus, there is an urgent need to identify novel interventions to target this population. Score-driving factors include: This application proposes to use a developed diabetes prevention program "Power to Prevent" via telehealth in a randomize trial of child/parent group versus the parent-only group to compare efficacy of program delivery. Using the African-American

family to provide support helps build self-efficacy and is innovative. However, the PL does not highlight the social-economic or institutional barriers beyond the individual study that contribute to the increased and disproportionate rates of obesity in the Mississippi Delta. Overall, this was a well-received application with some weakness that could be addressed in the future.

1.Significance:

Strengths

- African American (AA) youth who are prediabetic with high risk factors are more likely to progress to having diabetes, a major public health concern.
- Research indicates that there is a lack of specific primary prevention intervention for African-American children; thus, there is an urgent need to identify novel interventions to target this population, a major strength of the project.

Weakness

- There are concerns regarding access to internet/or the digital divide for the population in question and it is unclear how this is addressed in the study's protocol, a moderate weakness.

2. Investigators:

Strengths

- There is opportunity for career advancement and publications for the lead investigator, with mention of future plans for extramural grant funding.
- The research team, collaborators, and mentors have the appropriate level of expertise to conduct the proposed project.

Weaknesses

- The lead investigator has not published in this area of research and has limited experience in conducting of this type of research. This is a minor weakness.

3. Innovation:

Strengths

- This project presents the opportunity to fill gaps in research on using a family-based telehealth approach to reduce health disparities related to diabetes in African-American children.
- The re-framing of the "Power to Prevent" into a telehealth format versus the face-to-face format, which helps reach a wider targeted population.

Weaknesses

- Considering the location in which the study will take place and African-American family make-up, the project lacks justification for the selection of "only one child" per child-parent enrolled, a minor weakness.
- The project does not provide alternative plans for families that may not have or lose internet access for telehealth, a moderate weakness.

4. Approach:

Strengths

- An RCT to evaluate the diabetes prevention program using a telehealth intervention that compares the efficacy of parent-and-child versus parent-only intervention approaches in African Americans is proposed.

Weaknesses

- There is an underappreciation of the role that social and economic circumstances and environment (living in a food desert, neighborhood walkability, etc.) might play in terms of achieving the primary outcome (reduction in BMI/weight loss) in African-American families at high risk for diabetes, a moderate weakness.
- Minor weakness: More clarity is needed on the burden of meeting times (11 1-hour meetings plus 3 assessment visits). What are the alternatives in place for those that may not have access to internet services to utilize tablets for conferencing?
- While African Americans are savvy with using some mHealth technologies, tablets, and mobile apps, the project lacks plans to educate individual using telehealth, especially from Electronic Medical Records (patient portal?).

5. Environment:

Strengths

- The environment at UMMC is beyond sufficient for the investigator to conduct this research, including accessibility to the University of Mississippi Medical Center, the UMMC's Telehealth Center, the Clinical Translational Research Center, and the Mississippi Center for Obesity.
- Strong institutional support and commitment, including offers to match funds in efforts to increasing the number of well-trained chronic disease researchers committed to studying vulnerable populations.

Weaknesses

- None noted.

Study Timeline (Specific to applications proposing clinical trials):

Strengths

- This project has abundant resources available, including the Mississippi Center for Clinical and Translational Research, the Obesity COBRE, and the UMMC's Telehealth Center of Excellence.

Weaknesses

- The time commitment required for using telehealth and incentives for this group is not appropriate.

Protections for Human Subjects:

Acceptable Risks and/or Adequate Protections

Data and Safety Monitoring Plan (Applicable for Clinical Trials Only):

Acceptable

Inclusion of Women, Minorities and Children: Applicable Only for Human Subjects research and not IRB Exemption #4.

- Sex/Gender: Distribution justified scientifically
- Race/Ethnicity: Distribution justified scientifically
- For NIH-Defined Phase III trials, Plans for valid design and analysis: Not applicable
- Inclusion/Exclusion of Children under 18: Including children <18 justified scientifically

Vertebrate Animals:

Not Applicable (No vertebrate animals)

Biohazards:

Not Applicable (No Biohazards)

Select Agents:

Not Applicable (No Select Agents)

Resource Sharing Plans:

Acceptable

- UMMC currently has two COBRE Centers (Perinatal and Obesity COBREs) and CTSA; thus, there appears to be some overlap in services that are shared and requested.

Authentication of Key Biological and/or Chemical Resources:

Acceptable

Budget and Period of Support:

Recommend as Requested

CRITIQUE 2

SCORE (1-9): 4

Overall Impact: Project 4 is a research project led by Abigail Gamble, Ph.D. (Assistant Professor of Preventive Medicine and Pediatrics) and co-investigator Desiree Pendergrass, M.D. (UMMC Pediatrician) for Years 1-3. The study will implement and evaluate a telehealth diabetes prevention (Power to Prevent) in children age 6-11 years and their parents over a 12-week period after an 11-week group-based videoconferences, and includes a 30-week follow up. Overall, the proposal is well-organized and well-written. The study addresses a very significant issue around childhood and adult obesity and the development of type 2 diabetes, both of which are as high or higher in prevalence in Mississippi compared to other US populations. Despite studies and lifestyle interventions, significant gaps remain. Although other RCTs have been done in T2DM populations, this study will take advantage of the existing Mississippi Diabetes Telehealth Network, which has been implemented for rural adults. The scientific premise for the study has been adequately addressed.

The investigators are well-suited to complete the study based on their qualifications and prior related experience. The present study will be tailored to assess the early efficacy of the TELE-GEN intervention targeting overweight or obese parents and their children using a theoretical approach

expected to positively change behavior around food intake and physical activity. The strategy to target the family unit including youth and their parents is the innovative aspect of the proposal, but the fact that other currently funded studies at the institution may also be targeting obesity and diabetes prevention in adults weakens the novelty.

The Family Lifestyle Approach is feasible and will include an informational session, problem solving, family goal setting, and reinforcement of behaviors. Aim 1 will consist of randomization of participants recruited from the pediatric primary care clinic in the Jackson Medical Mall to obtain preliminary data of the comparative efficacy of two intervention (parent-and-child, or parent-only; N=20 families each) arms and appropriate baseline measures will be obtained. Aim 2 will determine the mediating effects of environmental and psychosocial variables on the differences in BMI and BMI z-score (child) as well as differences in BMI, A1c, waist circumference and other measures. There is adequate scientific rigor related to statistical analysis and power analysis to support the successful completion of the study. The timeline is well described. The protection for human subjects, inclusion/exclusion criteria, and data and safety monitoring are also well described. However, the recruitment plan is only briefly described. The Project Lead needs to provide more detail on the need for a replacement Project Lead. The timeline is well described. The environment is excellent and appropriate for the study. There is adequate description of protection of human subjects, in addition to inclusion/exclusion criteria and data and safety monitoring.

Overall, the proposal is well organized and well written, and enthusiasm for the successful completion of the study is high. It is believed that, given the large rural population, the telehealth intervention approach and basis of the study should contribute to a positive impact on this significant issue and advance the prevention of diabetes in this vulnerable population.

Protections for Human Subjects:

Acceptable Risks and/or Adequate Protections

- Well described.

Data and Safety Monitoring Plan (Applicable for Clinical Trials Only):

Acceptable

- o Well described.

Inclusion of Women, Minorities and Children: Applicable Only for Human Subjects research and not IRB Exemption #4.

- Sex/Gender: Distribution justified scientifically
- Race/Ethnicity: Distribution justified scientifically
- For NIH-Defined Phase III trials, Plans for valid design and analysis: Not applicable
- Inclusion/Exclusion of Children under 18: Including ages <18; justified scientifically
- Well justified.

Vertebrate Animals:

Not Applicable (No Vertebrate Animals)

Biohazards:

Not Applicable (No Biohazards)

Select Agents:

Not Applicable (No Select Agents)

Resource Sharing Plans:

Acceptable

- Well described.

Authentication of Key Biological and/or Chemical Resources:

Not Applicable (No Relevant Resources)

Budget and Period of Support:

Recommend as Requested

THE FOLLOWING SECTIONS WERE PREPARED BY THE SCIENTIFIC REVIEW OFFICER TO SUMMARIZE THE OUTCOME OF DISCUSSIONS OF THE REVIEW COMMITTEE, OR REVIEWERS' WRITTEN CRITIQUES, ON THE FOLLOWING ISSUES:

PROTECTION OF HUMAN SUBJECTS: ACCEPTABLE

INCLUSION OF WOMEN PLAN: ACCEPTABLE G1A

INCLUSION OF MINORITIES PLAN: ACCEPTABLE. Although the overwhelming majority of participants in the proposed studies will be African-American, Project 2 indicates that possibly 1% of participants will not be African American. Therefore, inclusion is coded as **M1A**.

INCLUSION OF CHILDREN PLAN: ACCEPTABLE C1A

COMMITTEE BUDGET RECOMMENDATIONS: The budget was recommended as requested.

SCIENTIFIC REVIEW OFFICER'S NOTES: The research projects are referenced inconsistently in the application. In the Overall component and Core 2, Project 2 is “Reducing the Risk of Cardiovascular Disease Among Breast Cancer Survivors in Medically Underserved Areas,” Project 3 is “Telehealth Diabetes Prevention Intervention for the Next Generation of African American Youth (TELE-GEN) study,” and Project 4 is “Reducing Pain-Related Disability and Opioid Use in Pediatric Sickle Cell Disease.” In the Admin Core, Cores 1 and 3, and the order of upload in the application, Project 2 is “Reducing Pain-Related Disability and Opioid Use in Pediatric Sickle Cell Disease,” Project 3 is “Reducing the Risk of Cardiovascular Disease Among Breast Cancer Survivors in Medically Underserved Areas,” and Project 4 is “Telehealth Diabetes Prevention Intervention for the Next Generation of African American Youth (TELE-GEN) study.” The reviewers were instructed to use the order of upload in the application for the project numbers.
