# Supplementary material for: Telehealth Diabetes Prevention Intervention for the Next Generation of African American Youth: Protocol for a Pilot Trial
Source: JMIR Res Protoc. 2021 Mar 31;10(3):e25699. doi: 10.2196/25699 (PMC8047807; doi:10.2196/25699)
Supplement: Multimedia Appendix 2 [file resprot_v10i3e25699_app2.pdf]

Project 4 - Telehealth Diabetes Prevention Intervention for the Next Generation of African American Youth (TELE-GEN Study)
PI: Gamble, Abigail, Ph.D.

DESCRIPTION (provided by applicant):
The Telehealth Diabetes Prevention Intervention for the Next Generation of African American Youth (TELE- GEN) study will evaluate the implementation and early efficacy of a telehealth diabetes prevention intervention for African American (AA) children (8- to 11-years-old) and their parents. Power to Prevent is a lifestyle diabetes prevention intervention from the Centers for Disease Control and

Prevention that is based on the Diabetes Prevention Program and tailored specifically for AA families. To our knowledge, this intervention has not yet been evaluated in a randomized trial with AA families with children at risk for T2DM, nor been delivered via telehealth. Employing an effectiveness-implementation hybrid research design, we aim to: (1) Conduct a pilot RCT to assess the (a) early efficacy of Power to Prevent delivered via telehealth to treat overweight/obesity in AA children and their parent, and (b) comparative efficacy of parent-and-child vs parent- only approaches. (2) Comprehensively evaluate a multifaceted implementation strategy for the uptake of Power to Prevent delivered via telehealth by UMMC's pediatric weight management clinic. Our primary outcome measure will be stabilization or reduction in BMI z-score in children (index participant) and a reduction in parent BMI (co-participant). Eligible overweight/obese children and their overweight/obese parent will be randomized to either a parent-and-child (n=20 families) or parent-only (n=20 families) intervention arm. Both arms will receive the same telehealth diabetes prevention intervention based on Power to Prevent and delivered by trained lifestyle coaches. Families in each intervention arm will meet weekly for 11-weeks (60-min sessions), and then monthly (60-min sessions) for 4 pilot behavioral reinforcement maintenance sessions (15 sessions total). Participants will meet in their respective groups (n=5 families per group) via videoconference using Wi-Fi-equipped tablets with cellular connectivity for the entire intervention. Sessions will consist of nutrition and physical activity education (20 min), problem solving and decision-making skills to circumvent barriers to behavioral change (20 min), and family goal setting and action planning (20 min). Assessment measures will be collected from the child and parent participants at baseline, 12-weeks (post-intervention), and 30-weeks (follow-up). The implementation strategy has two targets: (1) UMMC's pediatric weight management clinical setting and clinical care team; and (2) overweight/obese pediatric patients and their overweight/obese parents referred to and engaged in intensive obesity treatment for the prevention of T2DM. The multifacted implementation plan includes four discrete strategies: (1) creating a new clinical team; (2) changing the service site; (3) intervening with families; and (4) assessing organizational readiness. Preliminary findings will uniquely position the investigative team to conduct a full-scale effectiveness-implementation hybrid research study to test the interventions effectiveness for T2DM prevention, while evaluating a refined implementation protocol.

CRITIQUE 1
SCORE: 4

Overall Impact: African American (AA) youth who are prediabetic with high-risk factors are more likely to progress to having diabetes. While the Diabetes Prevention Program has demonstrated the efficacy of a lifestyle intervention to prevent Type 2 diabetes mellitus (T2DM) in adults through dietary and physical activity changes that lead to weight loss, there is no gold standard lifestyle intervention for the prevention of youth-onset T2DM, especially African American (AA) children. Thus, there is an urgent need to identify novel interventions to target this population. The proposed study will implement and evaluate a lifestyle, diabetes prevention intervention that aims to target obesity in AA youth at risk for T2DM and their parents. This application proposes to use [1] a effectiveness-implementation hybrid approach to evaluate a telehealth lifestyle intervention and [2] leverages an existing diabetes prevention program via Telehealth using an randomize trial of AA child/parent group versus the AA parent-only group to compare efficacy and implementation of program delivery, major strengths. A few weaknesses were noted, including the coverage of data usage for the Telehealth intervention and consideration of sex as biological variable. Overall, the proposed project has the potential to fill gaps in knowledge and accelerate evidence and translate research into routine practice.

1.Significance:
Strengths

- A major strength is the purposed research targets African American youth and their families who are disproportionately burden with obesity and are at high risk for type 2 diabetes.
- To date, there is a lack of standards to address the primary prevention of T2DM for youth. This study incorporates a telehealth intervention approach to address barriers such as access to health care in rural underserved area where African Americans have higher prevalence of obesity.
- This project presents the opportunity to fill gaps in research by using a family-based telehealth approach to reduce health disparities related to diabetes in African-American children, by re-framing the "Power to Prevent" into a telehealth format versus the face-to-face format, which helps reach a wider targeted population

Weaknesses

- None noted.

2. Investigators:

Strengths

- The Project Lead, Abigail Gamble, Ph.D. is an Assistant Professor of Preventive Medicine and Pediatrics, and the Director of Center for Child Health Equity, Myrlie Evers-Williams Institute for Elimination of Health Disparities, University of Mississippi Medical Center.
- Dr. Gamble, research collaborators, and mentors has the appropriate level of expertise and oversight to support project aims.
- Some of the members of the team have a successful history of collaboration.

Weaknesses

- No weakness noted.

3. Innovation:

Strengths

- This study explores an effectiveness-implementation hybrid research approach to prevent diabetes in a high-risk, African American pediatric population while simultaneously evaluating the feasibility and scalability of family-based intervention in a pediatric clinical setting
- The proposal leverages an existing diabetes prevention intervention (Power to Prevent) via Telehealth to promote diabetes prevention in African Americans using a parent-child dyad.

Weaknesses

- None noted.

4. Approach:

Strengths

- Examines the impact of a Telehealth intervention on T2DM prevention in African Americans families.
- The research design, an effectiveness-implementation hybrid research approach, is well reasoned and appropriate for the planned research.

- The study is guided by the integration of three theories: Family Systems Theory, the Social Learning Theory, and the Telehealth in Chronic Disease that are aligned with measures and outcomes of the proposed study.
- Considers family structure of African Americans and include block randomization method for selection and enrollment of participants.
- Address connectivity issues by providing Wi-Fi enabled tablets with cellular connectivity to mitigate digital barriers in terms of owning a device.

Weaknesses

- Sex as a biological variable is not considered. Also, it's unclear which measures capture or assess social determinates of health which may impact engagement in the intervention.
- While the study has considered an alternative for African American families who may not have internet access by providing Wi-Fi enabled tablets with cellular connectivity, there lack plans who will cover data usage fees.

5. Environment:

Strengths

- The environment is suitable and beyond sufficient for Project Leader to conduct the proposed research, including having access to and collaboration with the Telehealth Centers of Excellence and the Mississippi Center for Obesity.

Weaknesses

- No weakness noted.

Study Timeline (Specific to applications proposing clinical trials):

Strengths

- The study time is both reasonable and appropriate for planned enrollment and study procedures.

Weaknesses

- None noted.

Protections for Human Subjects:

Acceptable Risks and/or Adequate Protections

Data and Safety Monitoring Plan (Applicable for Clinical Trials Only):

Acceptable

Inclusion of Women, Minorities and Individuals Across the Lifespan (Applicable Only for Human Subjects research and not IRB Exemption #4)

- Sex/Gender: Distribution justified scientifically
- Race/Ethnicity: Distribution justified scientifically
- For NIH-Defined Phase III trials, Plans for valid design and analysis:
- Inclusion/Exclusion Based on Age: Distribution justified scientifically

Vertebrate Animals:
Not Applicable (No Vertebrate Animals)

Biohazards:
Not Applicable (No Biohazards)

Select Agents:
Not Applicable (No Select Agents)

Resource Sharing Plans:
Acceptable

Budget and Period of Support:
Recommend as Requested

CRITIQUE 2
SCORE: 4

Overall Impact: This ambitious project addresses a highly significant issue of pediatric obesity and the fact that the majority of cases of T2DM in children is due to obesity. This is particularly a problem in African American children. The researcher has some experience in this area and has assembled a good team of investigators and will get excellent support from the two proposed cores. Her study aims to conduct a pilot RCT to assess the efficacy of a CDC developed intervention, Power to Prevent, for African American families with obesity delivered via tele-health for Parent only vs. parent plus child to prevent T2DM. It is unclear what the control arm of the study will be or even if this intervention can be delivered successfully via telehealth vs. in person. It seems like she is trying to jump over many steps to get to a clinically useful intervention, and while this is admirable and such interventions are desperately needed, the study as proposed is problematic.
There are a number of issues:

1. Power to Prevent has not been tested in an RCT to date and therefore its efficacy needs to be tested the way it was intended to be used or testing the usual clinical obesity treatment care for these families vs. the telehealth intervention. As it is currently designed there is no control group so cannot call it an RCT.

2. If this is a prevention study for T2DM, then endpoints need to be insulin resistance, HA1C, or GTT as weigh loss may not be as important as improving metabolic insulin resistance.

3. If this is an obesity treatment study to prevent T2DM then in a 12-week intervention it is highly unlikely to see change in BMI z-score and BMI as the body composition and biometrics/biomarkers for obesity change much more quickly than BMI. So, use of BMI or BMI z score, no matter the sample size is unlikely to show a significant effect. Using dual-energy X-ray absorptiometry (DEXA), would be much more likely to show the possible changes in body composition given the short intervention timeline. Also fasting lipids (particularly triglycerides) and insulin level will change quickly in response to diet and exercise treatment goals. 5% weight loss in three months needs to be shown in their clinic population first as gold standard if she is claiming that this may be possible with her tele-health intervention.

4. The hybrid design of effectiveness/implementation design is a novel plan, but several aspects of this study need to be piloted first, including if the Tel-Gen works vs. standard care for such families. It doesn't seem feasible to have such a hybrid design without at least the Power to Prevent component having been tested and proven efficacious.

1.Significance:

Strengths

- Highly significant problem
- Important age group 8-13

Weaknesses

- No preliminary work in the clinic noted, even in response to prior review.
- Lack understanding of barriers to diet and exercise change such as poverty, transportation, violence, trauma as critical in understanding and managing pediatric overweight/obesity.
- Issue with some children entering puberty and others not so confounds group

2. Investigators:

Strengths

- Good collaborators and use of cores
- Good potential for this junior investigator

Weaknesses

- Why the additional work done during the revision year was not discussed weakens enthusiasm for this project and Project Leader.

3. Innovation:

Strengths

- Use of obesity treatment clinic, use of group visits, and engagement of parents are all standard of case in pediatric obesity treatment so capitalizing on these strengths in the Medical school are good
- Telehealth group visits may be innovative in this population

Weaknesses

- Not highly innovative
- Could benefit from use of DEXA, biomarkers, better dietary instruments

4. Approach:

Strengths

- Important topic in a high-risk population with an excellent choice of age range
- Telehealth is critical for these populations and will be even more critical going forward, good support from the Telehealth Center on campus

Weaknesses

- Unclear why this is being called an RCT without a control group
- Unclear why a parents-only approach is being taken as one of the study arms when the applicant clearly states that obesity treatment literature is clear on need for family approach
- Unclear why testing an unproven intervention Power to Prevent in a telehealth and not using another intervention that has been RCT tested
- Use of BMI and BMI z with short-term intervention without justification that this will show an efficacy, and many trials showing that BMI is extremely difficult to show change.
- Effectiveness-implementation hybrid research design not well justified and too premature for this work and this Project Leader
- Block Food Frequency Questionnaires (FFQ's) are difficult to use and would be better to use 24 hr diet recalls at least in a subset
- Percent body fat and fat free mass is calculated using skinfolds and can be highly inaccurate- need to carefully consider if these are useful. Much better to use DEXA.
- Human subjects leave out adults

5. Environment:

Strengths

- Excellent support from two proposed cores, good access to population of interest and clinical obesity treatment center.

Weaknesses

- Not taking advantage of biomarkers and DEXA scanning as endpoints when these are available and patients are coming to the medical center for assessments.

Study Timeline (Specific to applications proposing clinical trials):

- Six months start-up time
- Use of clinic and other resources to maximize recruitment

Weaknesses

- R01 proposed end year 3 not year 2 due to ambitiousness of study design

Protections for Human Subjects:

Unacceptable Risks and/or Inadequate Protections

- Recruitment and protection well described but forgot to add adults in sample

Data and Safety Monitoring Plan (Applicable for Clinical Trials Only):

Unacceptable

- o Again, unclear if DSMP is proposed with IAC or not

Inclusion of Women, Minorities and Individuals Across the Lifespan (Applicable Only for Human Subjects research and not IRB Exemption #4)

- Sex/Gender: Distribution justified scientifically
- Race/Ethnicity: Distribution justified scientifically
- For NIH-Defined Phase III trials, Plans for valid design and analysis: Scientifically acceptable
- Inclusion/Exclusion Based on Age: Distribution justified scientifically

- Children and adults well justified scientifically

Vertebrate Animals:
Not Applicable (No Vertebrate Animals)

Biohazards:
Not Applicable (No Biohazards)

Resubmission:

- Socioeconomic barriers to the study are not addressed and referrals to EverCare for vulnerable families is not a sufficient answer to the significant issues with making lifestyle changes for socioeconomically vulnerable families.
- Telehealth technology package and support now well-described
- Detailed recruitment plan by HSRC core well-described now
- How this project may interface with the obesity focused CTR and other projects is still unclear.
- Project Leader is still very inexperienced in this field and would benefit from additional clinic time and a less ambitious aim 1.

Select Agents:
Not Applicable (No Select Agents)

Resource Sharing Plans:
Acceptable

Authentication of Key Biological and/or Chemical Resources:
Not Applicable (No Relevant Resources)

CRITIQUE 3
SCORE: 5

Overall Impact: This is a pilot study to prepare for a true RCT in order to evaluate the efficacy of the Power to Prevent intervention that is suggested by the CDC in order to prevent the development of Type 2 diabetes. The goal of the intervention is to 'achieve 5% weight loss through dietary and physical activity modifications that are self-selected and self-regulated by participants, and by enhancing family

communication'. In this study it is proposed that they will not include a control group. Instead, the Project Leader proposes that they plan to test the intervention impact by comparing the effect in two groups; parent only and parent-child pairs. The primary outcome of study will be the difference in BMI z-score (child) and BMI (parent) change from baseline to 30 weeks across each arm. It is unclear why there is not a control group being studied or if appropriate comparative effective methods would be employed. The study provides the potential to make a significant impact on health outcomes by providing baseline information that could lead to the design of a future study. Additionally, it could provide an important training opportunity for the Project Leader to conduct research that could lead to future impactful studies.

The following are some points that should be considered regarding the study:

1. There is mention that the Biostatistics core will be accessed for analysis in the proposal for this project and there are not any biostatisticians or analysts budgeted for. There is very little indication on the Dr. Gamble's Biosketch that she has the appropriate training to conduct the analysis for a clinical trial and it is unclear which parts of the analysis she will be conducting herself. In a letter of support from Dr. Granger they state that the BERD Core will be accessible to Project Leaders and there is mention of this access in the strategy. This is a strength if they are able to provide support/collaboration, but specifics about how this would work should have been specified in the research strategy, timeline, and/or project budget. The lack of a control group does emphasize the need for specific biostatistics expertise.

2. A DSMB should be considered for this study and is related to point 1. Development of this team would also provide appropriate training for the Project Lead that will be needed for larger studies. Core 1 does state that " Training will also address the use of Data Safety and Monitoring Plans (DSMPs) and Data Safety and Monitoring Boards (DSMBs) in clinical research, in conjunction with the MCCTR Research Service Center." However, Dr. Gamble asserts it is not necessary.

3. Power calculations were completed but details of how this was calculated nor justification for the effect sizes studied were not provided.

4. It is unclear why someone with expertise in qualitative data analysis is not being resourced for this work. The qualitative work should be conducted or mentored/overseen by someone with expertise in that area.

5. There should be intent to conduct current or future study to evaluate the impact of telehealth compared to in person interventions. There are qualitative interviews being done in this study to understand how the telehealth intervention impacted the outcomes which will serve as a good background information to study this information later.

6. Data collection will be completed using REDCap which seems appropriate.

Additional Comments to Applicant (Optional)

- In future studies wearable sensors could be used to monitor and motivate compliance with physical activity portions of the intervention.

MEETING ROSTER

National Institute of General Medical Sciences Special Emphasis Panel
NATIONAL INSTITUTE OF GENERAL MEDICAL SCIENCES
Review of Centers of Biomedical Research Excellence (COBRE) P20 Applications

ZGM1 RCB-3 (C1)
07/16/2020 - 07/17/2020

Notice of NIH Policy to All Applicants: Meeting rosters are provided for information purposes only. Applicant investigators and institutional officials must not communicate directly with study section members about an application before or after the review. Failure to observe this policy will create a serious breach of integrity in the peer review process, and may lead to actions outlined in NOT-OD-14-073 at https://grants.nih.gov/grants/guide/notice-files/NOT-OD-14-073.html and NOT-OD-15-106 at https://grants.nih.gov/grants/guide/notice-files/NOT-OD-15-106.html, including removal of the application from immediate review.

CHAIRPERSON(S)

LOCKMAN, PAUL R., PHD
PROFESSOR AND THE DOUGLAS GLOVER ENDOWED CHAIR
DEPARTMENT OF BASIC PHARMACEUTICAL SCIENCES
SCHOOL OF PHARMACY
ASSOCIATE DIRECTOR FOR TRANSLATIONAL RESEARCH
WEST VIRGINIA UNIVERSITY HEALTH SCIENCES CENTER
MORGANTOWN, WV 79106

MEMBERS

ADAMS, ALEXANDRA K., MD, PHD
DIRECTOR AND PRINCIPAL INVESTIGATOR
CENTER FOR AMERICAN INDIAN AND
RURAL HEALTH EQUITY (CAIRHE)
MONTANA STATE UNIVERSITY
BOZEMAN, MT 59717

BARRETT, NADINE, PHD
ASSISTANT PROFESSOR, DIRECTOR
STAKEHOLDER ENGAGEMENT CORE
DEPARTMENT OF FAMILY MEDICINE AND COMMUNITY HEALTH
DUKE SCHOOL OF MEDICINE
DUKE UNIVERSITY
DURHAM, NC 27705

BHATNAGAR, ARUNI, PHD
PROFESSOR
DIVISION OF CARDIOVASCULAR MEDICINE
DIABETES AND OBESITY CENTER
DEPARTMENT OF MEDICINE
UNIVERSITY OF LOUISVILLE
LOUISVILLE, KY 40202

CUNNINGHAM, LEE ANNA, PHD
PROFESSOR
DEPARTMENT OF NEUROSCIENCE
UNIVERSITY OF NEW MEXICO
ALBUQUERQUE, NM 87131

DREW, KELLY L., PHD
PROFESSOR
DEPARTMENT OF CHEMISTRY AND BIOCHEMISTRY
INSTITUTE OF ARCTIC BIOLOGY
UNIVERSITY OF ALASKA-FAIRBANKS
FAIRBANKS, AK 99775

DURBIN, MATTHEW D., MD
ASSISTANT PROFESSOR
DEPARTMENT OF CLINICAL PEDIATRICS
INDIANA UNIVERSITY SCHOOL OF MEDICINE
INDIANAPOLIS, IN 46202

EDWARDS, DAVID G., PHD
PROFESSOR
DEPARTMENT OF KINESIOLOGY
AND APPLIED PHYSIOLOGY
UNIVERSITY OF DELAWARE
NEWARK, DE 19716

FITZGERALD, TRACY S., PHD
STAFF SCIENTIST, DIRECTOR
MOUSE AUDITORY TESTING CORE FACILITY
DIVISION OF INTRAMURAL RESEARCH
NATIONAL INSTITUTE ON DEAFNESS AND
OTHER COMMUNICATION DISORDERS (NIDCD)
BETHESDA, MD 20892

FUCHS, PAUL A., PHD
JOHN E BORDLEY PROFESSOR AND VICE CHAIR
DEPARTMENT OF OTOLARYNGOLOGY
HEAD AND NECK SURGERY
JOHNS HOPKINS UNIVERSITY
BALTIMORE, MD 21205

GEIGER, JONATHAN DAVID, PHD
CHESTER FRITZ DISTINGUISHED PROFESSOR
DEPARTMENT OF BIOMEDICAL SCIENCES
SCHOOL OF MEDICINE AND HEALTH SCIENCES
UNIVERSITY OF NORTH DAKOTA
GRAND FORKS, ND 58203

GRANDE, JOSEPH PETER, PHD, MD
PROFESSOR
DEPARTMENT OF LABORATORY MEDICINE
AND PATHOLOGY
MAYO CLINIC
ROCHESTER, MN 55905

HARRIS, NEIL, PHD
PROFESSOR
DEPARTMENT OF NEUROSURGERY
DAVID GEFFEN SCHOOL OF MEDICINE
UNIVERSITY OF CALIFORNIA, LOS ANGELES
LOS ANGELES, CA 90095

HE, JIANG, DSC, MD, PHD
PROFESSOR AND CHAIR
DEPARTMENTS OF EPIDEMIOLOGY AND MEDICINE
SCHOOL OF PUBLIC HEALTH AND TROPICAL MEDICINE
TULANE UNIVERSITY
NEW ORLEANS, LA 70112

KAPUSTA, DANIEL R., PHD
PROFESSOR
DEPARTMENT OF PHARMACOLOGY
LOUISIANA STATE UNIVERSITY HEALTH SCIENCES CENTER
NEW ORLEANS, LA 70112-1393

MALYKHINA, ANNA P., PHD
ASSOCIATE PROFESSOR
DIVISION OF UROLOGY
DEPARTMENT OF SURGERY
SCHOOL OF MEDICINE
UNIVERSITY OF COLORADO
AURORA, CO 80045

MARGULIES, KENNETH BER, MD
PROFESSOR
DEPARTMENT OF MEDICINE
UNIVERSITY OF PENNSYLVANIA SCHOOL OF MEDICINE
PHILADELPHIA, PA 19104

MC CARSON, KENNETH E., PHD
PROFESSOR
DEPARTMENT OF PHARMACOLOGY, TOXICOLOGY
AND THERAPEUTICS
UNIVERSITY OF KANSAS MEDICAL CENTER
KANSAS CITY, KS 66160

NICOLSON, TERESA A., PHD
PROFESSOR
DEPARTMENT OF OTOLARYNGOLOGY
HEAD AND NECK SURGERY
STANFORD UNIVERSITY
STANFORD, CA 924305

OH, EDWIN C., PHD
ASSOCIATE PROFESSOR
DEPARTMENT OF MEDICINE
NEVADA INSTITUTE OF PERSONALIZED MEDICINE
UNLV SCHOOL OF MEDICINE
LAS VEGAS, NV 89154

POMANN, GINA-MARIA, PHD
ASSISTANT PROFESSOR
DIRECTOR, DUKE CTSI BIOSTATISTICS, EPIDEMIOLOGY
AND RESEARCH DESIGN (BERD) METHODS CORE
DUKE CLINICAL AND TRANSLATIONAL SCIENCE INSTITUTE
DUKE UNIVERSITY
DURHAM, NC 27710

PUTLURI, NAGIREDDY, PHD
ASSOCIATE PROFESSOR
METABOLOMICS CORE
DEPARTMENT OF MOLECULAR AND CELL BIOLOGY
BAYLOR COLLEGE OF MEDICINE
HOUSTON, TX 77030

RIMOLDI, JOHN M., PHD
PROFESSOR
DEPARTMENT OF BIOMOLECULAR SCIENCES
SCHOOL OF PHARMACY
UNIVERSITY OF MISSISSIPPI
UNIVERSITY, MS 38677

RIVAS-TUMANYAN, SONA, DMD, DPH
ASSOCIATE PORFESSOR
OFFICE OF ASSISTANT DEAN FOR RESEARCH AND
THE DEPARTMENT OF SURGICAL SCIENCES
U OF PUERTO RICO SCHOOL OF DENTAL MEDICINE
SAN JUAN, PR 00936

ROUNDS, SHARON IRENE SMITH, MD
PROFESSOR
DEPARTMENT OF MEDICINE
BROWN MEDICAL SCHOOL
BROWN UNIVERSITY
PROVIDENCE, RI 02908

STEPHENS, JACQUELINE M., PHD
PROFESSOR
DEPARTMENT OF BIOLOGICAL SCIENCES
LOUISIANA STATE UNIVERSITY
BATON ROUGE, LA 70803

STILL, CAROLYN HARMON, PHD
ASSISTANT PROFESSOR
FRANCES PAYNE BOLTON SCHOOL OF NURSING
CASE WESTERN RESERVE UNIVERSITY
CLEVELAND, OH 44106

SWEDELL, JENNIFER
ASSOCIATE PRINCIPAL
SR. LABORATORY PLANNER
CO ARCHITECTS
LOS ANGELES, CA 90036

TYKOCKI, NATHAN ROGER, PHD
ASSISTANT PROFESSOR
DEPARTMENT OF PHARMACOLOGY & TOXICOLOGY
MICHIGAN STATE UNIVERSITY
EAST LANSING, MI 48824

WEBSTER, MICHAEL A., PHD
PROFESSOR
DEPARTMENT OF PSYCHOLOGY
UNIVERSITY OF NEVADA, RENO
RENO, NV 89557

WHITE, CARL, PHD
ASSOCIATE PROFESSOR
DEPARTMENT OF PHYSIOLOGY & BIOPHYSICS
CHICAGO MEDICAL SCHOOL
ROSALIND FRANKLIN UNIVERSITY
OF MEDICINE AND SCIENCE
NORTH CHICAGO, IL 60064

WILLETTE, AURIEL, PHD
ASSISTANT PROFESSOR
DEPARTMENT OF FOOD SCIENCE AND HUMAN NUTRITION
IOWA STATE UNIVERSITY
AMES, IA 50010

WILSON, TONY W., PHD
ASSOCIATE PROFESSOR AND VICE CHAIR OF BASIC AND
TRANSLATIONAL RESEARCH
DEPARTMENT OF NEUROLOGICAL SCIENCES
UNIVERSITY OF NEBRASKA MEDICAL CENTER
OMAHA, NE 68198

SCIENTIFIC REVIEW OFFICER

SIDOROVA, NINA, PHD
SCIENTIFIC REVIEW OFFICER
OFFICE OF SCIENTIFIC REVIEW
NATIONAL INSTITUTE OF GENERAL MEDICAL SCIENCES
NATIONAL INSTITUTES OF HEALTH
BETHESDA, MD 20892-6200

EXTRAMURAL SUPPORT ASSISTANT

MENDOZA, DOUGLAS E.
GRANTS TECHNICAL ASSISTANT
NATIONAL INSTITUTES OF HEALTH
OFFICE OF SCIENTIFIC REVIEW
45 CENTER DRIVE
3AN12
BETHESDA, MD 20892

Consultants are required to absent themselves from the room during the review of any application if their presence would constitute or appear to constitute a conflict of interest.
